# An exciton-polariton laser based on biologically produced fluorescent protein

**DOI:** 10.1126/sciadv.1600666

**Published:** 2016-08-19

**Authors:** Christof P. Dietrich, Anja Steude, Laura Tropf, Marcel Schubert, Nils M. Kronenberg, Kai Ostermann, Sven Höfling, Malte C. Gather

**Affiliations:** 1 Scottish Universities Physics Alliance (SUPA), School of Physics and Astronomy, University of St Andrews, North Haugh, St Andrews, Fife KY16 9SS, UK.; 2 Technische Physik, Wilhelm Conrad Röntgen Research Center for Complex Material Systems, Universität Würzburg, Am Hubland, 97074 Würzburg, Germany.; 3 Institut für Genetik, Technische Universität Dresden, 01062 Dresden, Germany.

**Keywords:** Polariton, bose-einstein, condensation, fluorescent protein, laser

## Abstract

Under adequate conditions, cavity polaritons form a macroscopic coherent quantum state, known as polariton condensate. Compared to Wannier-Mott excitons in inorganic semiconductors, the localized Frenkel excitons in organic emitter materials show weaker interaction with each other but stronger coupling to light, which recently enabled the first realization of a polariton condensate at room temperature. However, this required ultrafast optical pumping, which limits the applications of organic polariton condensates. We demonstrate room temperature polariton condensates of cavity polaritons in simple laminated microcavities filled with biologically produced enhanced green fluorescent protein (eGFP). The unique molecular structure of eGFP prevents exciton annihilation even at high excitation densities, thus facilitating polariton condensation under conventional nanosecond pumping. Condensation is clearly evidenced by a distinct threshold, an interaction-induced blueshift of the condensate, long-range coherence, and the presence of a second threshold at higher excitation density that is associated with the onset of photon lasing.

## Teaser

Microcavities filled with biologically produced green fluorescent protein show polariton condensation at room temperature.

## INTRODUCTION

Strong light-matter coupling occurs when interactions between the exciton reservoir of an active medium and a surrounding confined light field—typically generated by an optical microcavity—are strong enough to result in the formation of bosonic coupled light-matter particles, known as cavity polaritons (*1*). Under appropriate conditions, these integer spin particles relax into a common ground state with a joint particle wave function—a process called polariton condensation (PC) (*2*, *3*). PC can occur at significantly lower thresholds than regular photon lasing (*4*, *5*) because no population inversion is required (*6*). Owing to their limited exciton binding energy, most inorganic semiconductor materials do not support PC at room temperature. By contrast, the Frenkel-type excitons found in organic materials are stable at room temperature (*7*) and show enormous coupling strengths [up to 1 eV (*8*)]. However, so far, PC of “organic polaritons” has only been reported for a handful of materials and has required optical pumping with ultrashort pulses (sub–10 ps) to achieve sufficient excitation density without excessive exciton-exciton annihilation (*9*–*11*). In addition, fabricating high-*Q* microcavities without damaging the organic emitter contained inside has necessitated elaborated device fabrication processes. Finally, the characteristic two-threshold behavior expected for a polariton laser (where the second threshold indicates the onset of photon lasing) has not been observed in organic devices so far.

Here, we demonstrate polariton lasing from simple laminated high-*Q* microcavities filled with the biologically produced enhanced green fluorescent protein (eGFP). The emission of these hybrid bioinorganic devices shows a clear two-threshold behavior, corroborating the buildup of a polariton condensate at low excitation densities and the onset of photon lasing (with simultaneous transition to the weak-coupling regime) at higher excitation densities. Both PC and photon lasing are achieved with conventional nanosecond excitation, eliminating the need for ultrashort pump pulses.

**Fig. 1:**
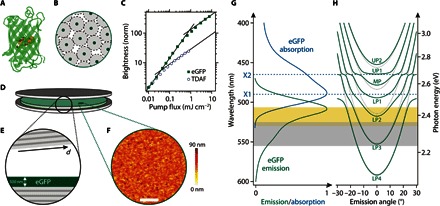
Laminated eGFP-filled high-*Q* microcavity used for polariton lasing. (**A**) Molecular structure of eGFP with the fluorophore in the center surrounded by 11 β sheets. (**B**) Schematic of how the β sheets prevent concentration quenching. (**C**) Normalized brightness versus pump fluence of thin films of solid-state eGFP and of the synthetic organic polariton material TDAF. Bimolecular exciton-exciton annihilation reduces spontaneous emission at high pump fluence (linear intensity increase changes to sublinear increase with slope 1/2). eGFP tolerates 20-fold higher pump fluence before the annihilation-induced sublinear behavior sets in. (**D** and **E**) Schematic illustration (D) and cross section of the microcavity containing a 500-nm-thick film of solid-state eGFP (E). The top mirror is slightly wedged with respect to the bottom mirror to allow adjustment of the cavity resonances by tuning the total cavity thickness *d*. (**F**) Atomic force microscopy topography map of the spin-coated GFP layer. Scale bar, 5 μm. (**G**) Absorption and emission spectrum of a solid-state eGFP film. The blue dashed lines indicate the two pronounced exciton resonances. The gray area marks the region over which gain is observed in eGFP [from the study by Dietrich *et al*. (23)], and the yellow region marks the range of polariton lasing (see Figs. 3 and 4). (**H**) Dispersion relation for uncoupled photonic modes (gray dotted lines) and strongly coupled polariton modes (green solid lines), calculated for typical cavity dimensions using a transfer matrix algorithm. UP, upper polariton branch; MP, middle polariton branch; LP, lower polariton branch.

There is growing interest in understanding how biomaterials affect light at the quantum level, for example, with respect to coherence and entanglement in photosynthetic complexes (*12*, *13*). Photosynthetic complexes are also studied as the active part of polariton devices (*14*). Fluorescent proteins (FPs) form a different class of biomaterial and, in the context of quantum biology, offer properties that are complementary to photosynthetic complexes. The discovery, cloning, and continued engineering of FPs (*15*–*18*) have revolutionized biomedical imaging, and FPs have become an essential tool in in vivo microscopy to label gene products and cellular components. Despite their widespread use, FPs were, until recently, used mainly in the spontaneous emission regime and in the aqueous, highly diluted environment inside cells. However, recent studies have shown that they are also interesting for stimulated emission (*19*–*21*). In addition, because of their unique molecular geometry, FPs are efficient solid-state fluorescent emitters, unlike many synthetic dyes (*22*–*24*). The FP molecule consists of a nanocylinder of 11 β sheets that enclose the actual fluorophore at the center of the molecule (Fig. 1A) (*25*, *26*). The β sheets act as a molecular bumper that ensures a 3- to 4-nm separation of fluorophores, even in the highly concentrated solid-state form of the material (Fig. 1B). This separation markedly slows down exciton diffusion and reduces concentration quenching (*27*). We hypothesized that the molecular bumper may also be beneficial to reduce exciton-exciton annihilation, which otherwise affects the performance of a gain medium at the high excitation levels required for lasing and PC.

## RESULTS AND DISCUSSION

### Suppression of exciton-exciton annihilation in eGFP

To investigate the performance of FPs at high exciton concentrations, we compared the fluorescence of thin films of eGFP and of 2,7-bis[9,9-di(4-methylphenyl)-fluoren-2-yl]-9,9-di(4-methylphenyl)fluorine (TDAF), one of the materials in which PC of organic polaritons was recently achieved (using femtosecond optical pumping) (*11*). When excited with low-intensity nanosecond light pulses, both films initially showed a linear increase in brightness with pump fluence (Fig. 1C). However, above a critical fluence of *E*_0,TDAF_ = 0.037 mJ/cm^2^, the brightness of the TDAF sample began to increase more slowly (∝ *E*^1/2^). This square root dependence is a fingerprint of bimolecular exciton-exciton annihilation being the dominant channel of exciton decay (*28*). The eGFP film does not show significant exciton-exciton annihilation up to a 20-fold higher pump fluence (*E*_0,eGFP_ = 0.80 mJ/cm^2^). Using the critical fluence for each material and a rate equation model, we estimate effective annihilation rate constants of *k*_XX,eGFP_ = 2.4 × 10^9^ s^−1^ and *k*_XX,TDAF_ = 6.1 × 10^10^ s^−1^ for eGFP and TDAF, respectively (section S1), indicating that the high exciton densities required for PC are achieved more readily with eGFP. The relatively low annihilation rate constant also implies that large exciton densities may be achieved in eGFP films using nanosecond, instead of picosecond or femtosecond, pumping.

### eGFP-filled laminated microcavities

We next fabricated laminated microcavities composed of an eGFP film (thickness, 500 nm) sandwiched between two dielectric Bragg mirrors (Fig. 1D). The lamination process yields a one-dimensional in-plane thickness gradient of ~1.5 to 4.5 μm (Fig. 1E), which allows us to tune the cavity resonances across a wide spectral range. The eGFP film has a root mean square roughness of 8.2 nm, as determined by atomic force microscopy (Fig. 1F). The absorption of a pristine protein film is dominated by two excitonic transitions located at 2.53 eV (490 nm, X1) and 2.67 eV (465 nm, X2), respectively (Fig. 1G). The emission spectrum peaks at 2.44 eV (508 nm). Both the absorption and emission spectrum are significantly broadened and show considerable overlap. Combined with the spectral and spatial overlap between cavity and photon modes, this overlap between emission and absorption is indispensable for effective energy transfer between photon and exciton and thus for PC. By contrast, large exciton linewidths have been considered as a hurdle to achieving condensation (*7*), and much effort has been directed toward finding organic materials with particularly narrow linewidths so that the polariton linewidth is smaller than the Rabi splitting of the coupled system (*10*). Here, we follow a different approach: By increasing the overall cavity thickness and coupling several cavity modes to the excitonic transitions of eGFP (Fig. 1H), we boost the *Q* factor of the involved cavity modes and increase the photonic character of the coupled cavity polaritons (86% photonic, section S2), which then also reduces the polariton linewidth.

**Fig. 2:**
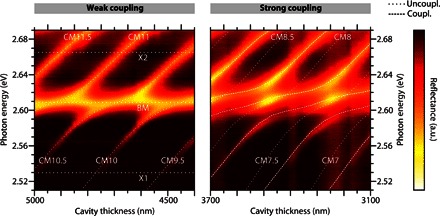
Experimentally obtained thickness-dependent reflectivity spectra (transverse electric–polarized) for eGFP-filled microcavities. Dotted lines represent uncoupled photon (CM*n*, BM) and exciton modes (X1 and X2). Dashed lines represent eigenvalues of the coupled oscillator matrix obtained from coupled harmonic oscillator calculations. Associated coupling strengths, *V*_1_ = 97 meV and *V*_2_ = 46 meV. In the weak-coupling regime (left, high water content), modes cross, whereas strong exciton-photon interaction (right, low water content) shows anticrossing of the involved modes. a.u., arbitrary units.

We found that the density of eGFP within the active region of the microcavity is crucial to realizing strong cavity-photon interaction. Reflectance measurements of microcavities containing eGFP with high and low water content (see Materials and Methods) are shown in Fig. 2. The spectra were recorded at the short-wavelength edge of the stop band of the dielectric mirrors to increase the visibility of the involved effects. As a consequence, the spectra show not only contributions of the cavity modes (CM*n*) but also the first Bragg mode (that is, Bragg minimum) (BM) of the dielectric mirrors. For the microcavity containing eGFP with high water content, the cavity modes and the Bragg mode overlap spectrally (at certain thicknesses). By contrast, a clear splitting occurs between all observed modes and for all thicknesses for the eGFP film with low water content. This avoided crossing is a clear signature of strong coupling between eGFP excitons and cavity modes. We attribute the difference between the two samples to the higher concentration of eGFP fluorophores in the film with low water content. In “traditional” polariton microcavities with only one exciton and one photon mode, anticrossing is expected between the upper and the lower polariton branch, as well as between the polariton branches and uncoupled exciton and photon modes. Only the latter is present here because of the multimodality of both the photonic and electronic systems. Fitting a coupled oscillator model to the experimental data for the low–water content sample yields coupling strengths of *V*_1_ = (97 ± 8) meV and *V*_2_ = (46 ± 5) meV for the X1 and X2 transitions, respectively (section S2).

### First threshold and polariton lasing

**Fig. 3:**
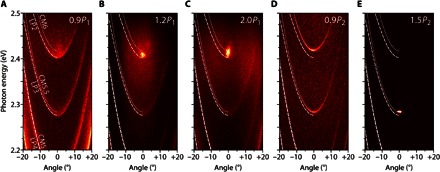
Excitation energy–dependent, angle-resolved luminescence spectra of low–water content eGFP-filled microcavity. (**A**) Excitation below polariton lasing threshold (*P* = 0.9*P*_1_). Expected position of lower polariton branches LP2, LP3, and LP4 (white dashed lines) and cavity modes CM5, CM5.5, and CM6 (white dotted lines) are shown. Note that emission is present along the polariton branches and, to a lesser extent, blueshifted from the dispersion minimum of the LP2 branch. (**B**) At excitation energies above *P*_1_, a distinct peak (attributed to the polariton condensate) emerges at an emission angle of 0° and is blueshifted relative to the minimum of the LP2 dispersion. (**C**) At *P* = 2*P*_1_, the blueshift of the condensate peak has increased further. (**D**) Around *P* = 9*P*_1_ = 0.9*P*_2_, the emission into the LP modes disappears and the polariton peak collapses. Instead, emission from the uncoupled photon modes CM*n* occurs. (**E**) At *P* = 1.5*P*_2_, a sharp emission peak appears at 0° from the CM5.5 mode (attributed to photon lasing).

**Fig. 4:**
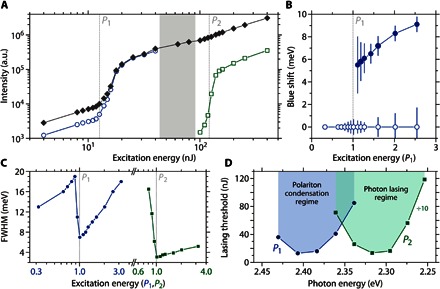
Analysis of condensate and photon laser properties. (**A**) Integrated microcavity emission intensity (filled diamonds), as well as individual contributions of LP2 (open circles) and CM5.5 mode (open squares) versus excitation energy. The two thresholds are attributed to the onset of polariton lasing (*P*_1_) and photon lasing (*P*_2_). (**B**) Spectral shift of the polariton condensate (closed symbols) and LP2 polariton branch (open symbols) as a function of the excitation energy (in units of *P*_1_). (**C**) Spectral width of the LP2 polariton emission (blue symbols) and the photon emission around CM5.5 (green symbols) as a function of excitation energy (in units of *P*_1_ and *P*_2_, respectively). FWHM, full width at half maximum. (**D**) Threshold energies of polariton lasing (*P*_1_) and photon lasing (*P*_2_) for different tuning of the cavity. Cavity tuning is achieved by scanning along the thickness gradient of the microcavity. *P*_2_ values were divided by 10 for clarity. Note the two distinct minima in threshold for the PC and photon lasing regime, respectively (2.40 and 2.31 eV).

We subsequently pumped the microcavities off-resonance (λ = 460 nm, close to the X2 band) using nanosecond pulses. Whereas the exciton lifetime of eGFP is in the few-nanosecond range (*22*), the lifetime of cavity photons is ≈200 fs (determined from the natural linewidth in a similar weakly coupled microcavity). This means that the LP2 polaritons have a sub-picosecond lifetime (≈250 fs) and therefore that the nanosecond excitation pulses effectively constitute a steady-state excitation of the system. Fourier imaging was used to record the angular-dependent microcavity emission at different excitation pulse energies (Fig. 3 and section S3). At low excitation energies (<10 nJ), emission was from the lower polariton branches LP*n* with strongly decreasing intensity toward higher emission angles, following a Boltzmann occupation along the polariton dispersion (section S4), thus showing the absence of any polariton bottleneck (*10*). When increasing the excitation energy, a critical point *P*_1_ = 12 nJ was reached, where the emission (i) shifted to higher energies, (ii) increased nonlinearly in intensity (see Fig. 4A), and (iii) drastically narrowed in linewidth (also see Fig. 4C), as well as in momentum spread around zero emission angle. This indicates the onset of PC by scattering of polaritons into the ground state of the LP2 dispersion. Furthermore, the polarization of the condensate is pinned to the polarization of the pump source (section S5). Energetically, the condensate lies well above the eGFP gain spectrum (*23*), which excludes conventional photon lasing as an explanation for these observations. Finally, we observed the formation of interference fringes at the condensation threshold (section S6). We attribute this to the presence of a collective macroscopic phase of polaritons with spatial coherence.

When increasing the pump energy further, the polariton condensate peak shifted even more into the blue (Fig. 4B, filled circles), which is a sign of nonlinear polariton-polariton and polariton-exciton interactions in the condensate. By contrast, the lower dispersion curve does not shift considerably with pump energy (Fig. 4B, open circles) for excitation powers up to 2.5 *P*_1_. This is further evidence for polariton lasing and against conventional photon lasing. (In the latter, frequency pulling of the lasing peak toward the gain maximum of eGFP would cause a redshift with increasing pump energy.) At the same time, the emission from the condensed cavity polaritons remains energetically below the expected dispersion of uncoupled cavity photons (Fig. 3, B and C, dotted line); the maximum blueshift of the condensate is (9.2 ± 1.0) meV, whereas the energy difference between the coupled and uncoupled dispersion minimum is (15.1 ± 0.5) meV.

### Second threshold and photon lasing

For excitation energies above 3*P*_1_, we observed a shift of the emission from the coupled polariton dispersion to the uncoupled cavity photon dispersion (Fig. 3D). We interpret this as the transition to the weak-coupling regime due to the depletion of the eGFP ground state. At a pump energy of *P*_2_ = 125 nJ, the microcavity emission began to again increase super-linearly with pump energy. This second threshold is an order of magnitude higher than *P*_1_ and is associated with the onset of photon lasing in the protein microcavity. Photon lasing is again accompanied by a decrease in linewidth (Fig. 4C), but there is no interaction-induced blueshift. Note that the photon-lasing peak does not originate from the dispersion minimum of the uncoupled cavity mode (CM6, around 2.42 eV) associated with the mode that showed PC but is strongly redshifted to the next, energetically lower uncoupled cavity mode (CM5.5, at around 2.28 eV). We attribute this to mode hopping into the cavity mode closest to the gain maximum of eGFP at around 2.25 to 2.3 eV (*23*). For the higher-energy cavity mode, the material gain is insufficient to achieve conventional photon lasing. This observation further supports our interpretation that the first lower threshold is associated with PC.

### Dependence of thresholds *P*_1_ and *P*_2_ on spectral detuning

Changing the detuning of a particular cavity mode with respect to the excitonic transitions (by scanning along the thickness gradient of the microcavity) gives insight into the wavelength dependence of the thresholds for polariton (*P*_1_) and photon (*P*_2_) lasing (Fig. 4D). We observed two bands with minima at 2.40 and 2.31 eV for *P*_1_ and *P*_2_, respectively. The *P*_2_ band is a qualitative measure of the eGFP gain spectrum and agrees well with our recent investigations (*23*). The *P*_1_ band clearly peaks outside of the gain region of eGFP, and the lowest PC threshold is an order of magnitude smaller than the lowest *P*_2_ value.

## CONCLUSION

The compatibility with nanosecond optical pumping and the simplicity of our lamination approach represent major improvements over previous organic polariton lasers. They bring PC and its remarkable properties, such as low-threshold coherent light generation and superfluidity, within easier reach and represent a step toward continuous-wave polariton lasing at room temperature. To the best of our knowledge, the two-threshold behavior observed here has not been reported for an organic system so far because of a combination of photobleaching and exciton-exciton annihilation at high excitation densities (*10*, *11*). The small Rabi splitting of our system, combined with the very weak bimolecular quenching, makes it much easier to fully deplete the ground state and thus leave the strong-coupling regime. The observation of polariton and photon lasing from one device thus demonstrates the robustness and efficiency of eGFP as an active optical material. In the future, cavities loaded with a combination of different FPs will enable controlled studies of energy transfer. By exploiting the complex interaction of FPs with water, we expect that solvent-assisted microshaping will yield complex intracavity patterns, paving the way to the study of macroscopic quantum interference effects in FP films.

## MATERIALS AND METHODS

### Sample preparation

The open reading frame of eGFP was polymerase chain reaction–amplified and cloned into vector pET23b (Novagen) using standard molecular biology methods (*29*). The corresponding protein contains an N-terminal hemagglutinin tag for immune detection and a His_6_ tag for protein purification. *Escherichia* *coli* strain BL21(DE3)pLysS (Novagen) was transformed with the construct using LB medium with ampicillin (100 μg/ml) and chloramphenicol (34 μg/ml) for selection. Cells were grown at 30°C until OD_600_ (optical density at 600 nm) of 0.5 to 0.6 and harvested 4 hours after protein expression was induced with isopropyl-β-d-thiogalactopyranoside (final concentration, 0.5 mM). Cells were resuspended in buffer [20 mM tris (pH 7.9), 500 mM NaCl, and 5 mM imidazole] and disrupted by sonification and treatment with lysozyme, and eGFP was purified using His•Bind Resin (Novagen). After elution, the protein-containing fraction was dialyzed against phosphate-buffered saline twice using a Slider-A-Lyzer Dialysis cassette (10K molecular weight cutoff, 15 ml) (Thermo Fisher Scientific). Subsequently, eGFP was filtered and centrifuged to remove buffer salts and increase protein concentration (to up to 220 g/liter). Concentrated solutions (~100 μl) were spin-coated (1000 rpm for 60 s) onto dielectric mirrors (distributed Bragg reflectors, surface roughness below λ/10) designed for peak reflectance (*R* ≥ 99.995%) at a wavelength of 532 nm and consisting of 14 pairs of alternating SiO_2_ (73-nm)/Ta_2_O_5_ (59-nm) layers. The structure was capped with an identical mirror on top to form the microcavity. Subsequently, the eGFP solution dried out and left a solid eGFP film with a nearly constant thickness of around 500 nm. Microcavities were characterized directly after spin coating and again after the drying process. In this way, the same sample with different water content of the eGFP film inside the cavity was investigated. The manual capping yielded a gradient in cavity thickness, spanning from 1500 to 4500 nm, which enabled scanning through different cavity modes by translating the sample. The theoretical quality factors of the photonic modes reach up to *Q* = 50,000. The thin films of eGFP for fluorescence characterization were fabricated on glass substrates in the same manner as described above. TDAF (Lumtec) was used as a representative example of a synthetic organic polariton laser material. Thin films of TADF were deposited by thermal evaporation under high vacuum.

### Cavity characterization

Reflectance measurements were performed with a custom-designed microscopic setup in epi-illumination and Fourier imaging configuration. A broadband white light-emitting diode was focused onto the sample surface through a high numerical aperture objective (NA = 0.42) covering an angular range of ±25°. The reflected light was collected through the same objective, dispersed by a 500-mm imaging spectrograph with a spectral resolution down to 0.03 nm, and imaged onto a cooled electron-multiplying charge-coupled device array detector. For measurements of the microcavity emission, the structure was excited by a pulsed, wavelength-tunable optical parametric oscillator system tuned to 460 nm (pulse length, 7 ns) using the same optical setup as for the reflectance measurements. The pump laser is slightly elliptic and was focused down to an 8-μm spot (average full width at half maximum) on the sample. The same system was also used for the fluorescence measurements of eGFP and TDAF on glass substrates.

### Matrix simulations

To model the experimentally observed dispersions of cavity photons and polaritons, and to determine the coupling strengths between eGFP excitons and cavity photons, we calculated the eigenstates of a coupled oscillator matrix that accounts for several cavity modes strongly interacting with two main excitonic transitions of the eGFP fluorophore. The matrix reads as follows

$$\left(\begin{array}{cccccc} E_{X2} & 0 & V_{2} & V_{2} & \cdots & V_{2}\\ 0 & E_{X1} & V_{1} & V_{1} & \cdots & V_{1}\\ V_{2} & V_{1} & E_{\text{Ph},1} & 0 & \cdots & 0\\ V_{2} & V_{1} & 0 & E_{\text{Ph},2} & \cdots & 0\\ \vdots & \vdots & \vdots & \vdots & \ddots & \vdots \\ V_{2} & V_{1} & 0 & 0 & 0 & E_{\text{Ph},n} \end{array}\right)$$

with the main exciton transition *E*_X1_ at 2.53 eV, its less pronounced sideband transition *E*_X2_ at 2.67 eV, and the uncoupled photon mode energies *E*_Ph,*n*_, as well as the coupling strengths *V*_1_ and *V*_2_ to the transitions *E*_X1_ and *E*_X2_, respectively. Because of the simultaneous coupling to different cavity modes CM*n* and the Bragg mode BM, we treat the light field as a nonpure state (superposition of different modes). For most spectra, we achieved a quantitative agreement between experiment and theory by taking 10 photon modes close to the exciton resonances into account. The uncoupled cavity dispersions were determined by transfer matrix calculations (section S2) using a background refractive index of 1.51 for the spin-coated eGFP thin film.

## Supplementary Material

http://advances.sciencemag.org/cgi/content/full/2/8/e1600666/DC1

## SUPPLEMENTARY MATERIALS

Supplementary material for this article is available at http://advances.sciencemag.org/cgi/content/full/2/8/e1600666/DC1 section S1. Analysis of pump fluence–dependent fluorescence measurements. section S2. Transfer matrix and coupled oscillator matrix calculations. section S3. Excitation-dependent zero-momentum emission of the eGFP microcavities. section S4. Thermalization of the polariton emission. section S5. Polarization of the condensate. section S6. Spatial coherence of the polariton condensate. fig. S1. Modeling of bimolecular exciton-exciton annihilation. fig. S2. Reflectance of a passive microcavity. fig. S3. Hopfield coefficients. fig. S4. Zero-momentum emission of eGFP-filled microcavity under nanosecond optical excitation. fig. S5. Energy dependence of occupation of the LP2 polariton branch for different excitation densities. fig. S6. Polarization pinning of the condensate. fig. S7. Spatial coherence of eGFP polariton condensates. References (*30*–*34*)
