# Supplementary material for: An exciton-polariton laser based on biologically produced fluorescent protein
Source: Sci Adv. 2016 Aug 19;2(8):e1600666. doi: 10.1126/sciadv.1600666 (PMC4991930; doi:10.1126/sciadv.1600666)
Supplement: http://advances.sciencemag.org/cgi/content/full/2/8/e1600666/DC1 [file supp_2_8_e1600666__index.html]

Science Advances | Science Advances

## Supplementary Materials

**This PDF file includes:**

- section S1. Analysis of pump fluence–dependent fluorescence measurements.
- section S2. Transfer matrix and coupled oscillator matrix calculations.
- section S3. Excitation-dependent zero-momentum emission of the eGFP microcavities.
- section S4. Thermalization of the polariton emission.
- section S5. Polarization of the condensate.
- section S6. Spatial coherence of the polariton condensate.
- fig. S1. Modeling of bimolecular exciton-exciton annihilation.
- fig. S2. Reflectance of a passive microcavity.
- fig. S3. Hopfield coefficients.
- fig. S4. Zero-momentum emission of eGFP-filled microcavity under nanosecond optical excitation.
- fig. S5. Energy dependence of occupation of the LP2 polariton branch for different excitation densities.
- fig. S6. Polarization pinning of the condensate.
- fig. S7. Spatial coherence of eGFP polariton condensates.
- References (*30*–*34*)

Download PDF

**Files in this Data Supplement:**

- Adobe PDF - 1600666\_SM.pdf
