# Supplementary material for: An exciton-polariton laser based on biologically produced fluorescent protein
Source: Sci Adv. 2016 Aug 19;2(8):e1600666. doi: 10.1126/sciadv.1600666 (PMC4991930; doi:10.1126/sciadv.1600666)
Supplement: http://advances.sciencemag.org/cgi/content/full/2/8/e1600666/DC1 [file 1600666_SM.pdf]

Science Advances

AAAS

advances.sciencemag.org/cgi/content/full/2/8/e1600666/DC1

Supplementary Materials for

An exciton-polariton laser based on biologically produced fluorescent protein

Christof P. Dietrich, Anja Steude, Laura Tropf, Marcel Schubert, Nils M. Kronenberg, Kai Ostermann, Sven Höfling, Malte C. Gather

Published 19 August 2016, *Sci. Adv.* **2**, e1600666 (2016)
DOI: 10.1126/sciadv.1600666

This PDF file includes:

- section S1. Analysis of pump fluence–dependent fluorescence measurements.
- section S2. Transfer matrix and coupled oscillator matrix calculations.
- section S3. Excitation-dependent zero-momentum emission of the eGFP microcavities.
- section S4. Thermalization of the polariton emission.
- section S5. Polarization of the condensate.
- section S6. Spatial coherence of the polariton condensate.
- fig. S1. Modeling of bimolecular exciton-exciton annihilation.
- fig. S2. Reflectance of a passive microcavity.
- fig. S3. Hopfield coefficients.
- fig. S4. Zero-momentum emission of eGFP-filled microcavity under nanosecond optical excitation.
- fig. S5. Energy dependence of occupation of the LP2 polariton branch for different excitation densities.
- fig. S6. Polarization pinning of the condensate.
- fig. S7. Spatial coherence of eGFP polariton condensates.
- References (*30–34*)

section S1. Analysis of pump fluence–dependent fluorescence measurements

Let n be the density of fluorophores in a thin film of fluorescent material, $n_1(t)$ the density of excited state fluorophores and $n_0(t) = n - n_1(t)$ the density of non-excited fluorophores. In general, the temporal evolution of n_1 can be described by

$$\frac{dn_1(t)}{dt} = \frac{I_p(t)\sigma}{h\upsilon_p} n_0(t) - kn_1(t) - \gamma n_1(t)^2 \qquad (1)$$

where $I_p(t)$ and υ_p are the intensity and frequency of the pump light, h is Planck's constant, σ is the absorption cross section of the fluorophores, $k = 1/\tau$ is the decay rate or inverse excited state lifetime of the fluorophore, and γ is the bi-molecular quenching constant.

We numerically solved Eq. 1 for a pump pulse with a Gaussian temporal profile, a FWHM pulse duration of t_p = 7 ns and pump fluences between 10^{-6} and 10^{-1} J/cm^2. The fluorescence intensity at each point in time is proportional to $n_1(t)$. Integrating $n_1(t)$ over the entire length of the pulse thus provides a measure of the total fluorescence generated. Figure 1 shows the dependence of $\int n_1(t)dt$ on pump fluence E. One can clearly distinguish the region of linear increase at lower fluence (here, emission is the dominant channel by which excitons decay) and a region with square root type increase at higher fluence (here, the dominant channel of exciton decay is through exciton-exciton annihilation). The pump fluence E_0 at which the slope changes is the point where the rates for radiative decay and exciton-exciton annihilation are equal, i.e.

$$kn_1(t) = \gamma n_1(t)^2 \qquad (2)$$

To estimate the exciton-exciton annihilation rate constant from E_0, we solved Eq. 1 analytically which can be readily done if one assumes constant pump light intensity, i.e. $I_p(t) = I_{p,0}$. The steady state limit of this analytic solution is given by

$$\lim_{t\to\infty} n_1(t) = \frac{1}{2\gamma}\left(\sqrt{\frac{4I_{p,0}\sigma}{h\upsilon_p}\gamma n + \left(\frac{I_{p,0}\sigma}{h\upsilon_p} + k\right)^2} - \left(\frac{I_{p,0}\sigma}{h\upsilon_p} + k\right)\right) \qquad (3)$$

Using the same argument about the fluorescence being proportional to $n_1(t)$ as above, $\lim_{t\to\infty} n_1(t)$ also provides a measure of the generated fluorescence. Furthermore, as shown in fig. S1, $\lim_{t\to\infty} n_1(t)$ derived from the analytical solution and $\int n_1(t)dt$ from the numerical solution show nearly identical dependence on pump fluence, if $I_{p,0} = E/t_p$ is taken as steady state pump light intensity. Taking $E = E_0$ and inserting Eq. 3 into Eq. 2, we can therefore obtain the effective annihilation rate constant k_{XX} of the material

$$k_{XX} = \gamma n = \frac{3}{4}\left(\frac{E_0\sigma}{2t_p h\upsilon_p}\right)^{-1}\left(\frac{E_0\sigma}{2t_p h\upsilon_p} + k\right)^2 \quad (4)$$

This expression only depends on the pump fluence E_0, at which exciton-exciton annihilation becomes dominant, on the wavelength and duration of the pump pulses and on the absorption cross section and excited state lifetime of the fluorophore. The latter two are known for eGFP and for the TDAF molecule studied here; they can also be measured relatively easily for most other materials. Using for eGFP, τ_{eGFP} = 3.3 ns and $\sigma(\lambda$=470 nm) = 2.1×10^{-16} cm^2 (Ref. (*22*)), we obtain $k_{XX,eGFP}$ = 2.4×10^9 s^{-1}. For the TDAF used as a reference, we have τ_{TDAF} = 0.89 ns and $\sigma(\lambda$=355 nm) = 1.4×10^{-15} cm^2 (Ref. (*29*)) giving $k_{XX,TDAF}$ = 6.1×10^{10} s^{-1}.

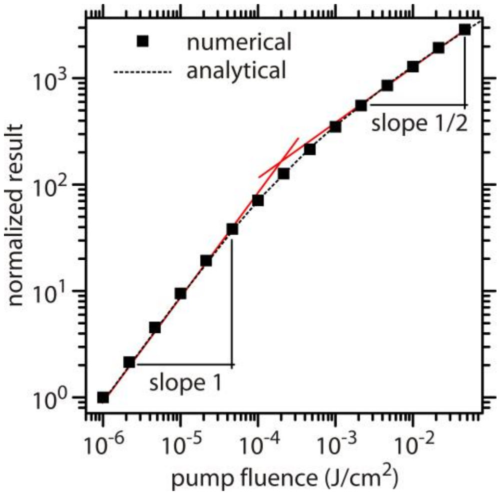

fig. S1. Modelling of bimolecular exciton-exciton annihilation. *Dependence of numerical solution of Eq. 1 (squares) and of analytical solution for steady state case (dashed black line) on pump fluence. Red lines are guides to the eye.*

section S2. Transfer matrix and coupled oscillator matrix calculations

In order to model the uncoupled photonic system formed by the highly reflective dielectric mirrors, transfer-matrix calculations of a microcavity filled with a passive, non-absorbing material were performed, i.e. the material had no excitonic contribution. The sample structure consisted of a 1mm-thick SiO_2 substrate with 14 pairs of alternating SiO_2 (73 nm) and Ta_2O_5 (59 nm) layers on top forming the bottom dielectric mirror, followed by a 500-nm-thick eGFP layer, an air gap with variable thickness and an identical dielectric mirror on top. The central wavelength of the mirrors was λ = 532 nm. The overall cavity thickness is the sum of the thicknesses of the eGFP layer and the gap above. The refractive index of eGFP was assumed to be $n_{eGFP} = 1.51$.

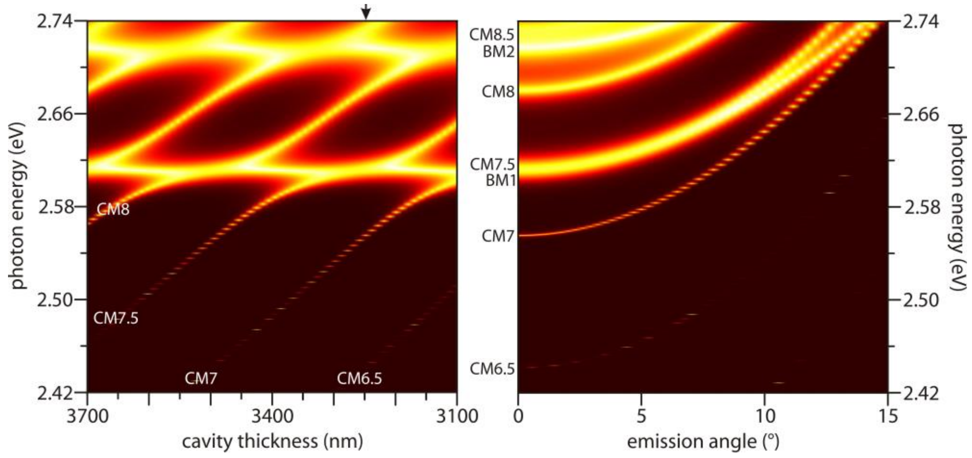

fig. S2. Reflectance of a passive microcavity. *Thickness- (left) and angular-dependent (right) calculated reflectance of a microcavity consisting of identical top and bottom dielectric mirrors (14 pairs SiO_2/Ta_2O_5, 532 nm central wavelength), a passive non-absorbing eGFP layer (500 nm thickness) and an air gap of varying thickness. The black arrow indicates the cavity thickness assumed for the angular-resolved reflectance map, d = 3240 nm.*

The reflectance of this structure was calculated as a function of the overall cavity thickness and the emission angle. The results are shown in fig. S2 (the thickness-dependent reflectance is shown for normal incidence). Cavity modes show mode quality factors up to Q = 50,000. By changing the cavity thickness, the spectral position of the confined cavity modes shifts across the stop band of the dielectric mirrors since the conditions for constructive interference change. By contrast, the Bragg modes of the dielectric mirrors are not subject to these shifts as their position is given by the structure of the mirror itself. Since the mirror distance is a multiple n of the central wavelength of the dielectric mirrors, fig. S2 shows several cavity modes with different mode numbers CMn. Note that cavity modes can have half-integer mode numbers since constructive interference between the dielectric mirrors arises for every $\lambda/2$.

The angular-dependent reflectance of the passive eGFP microcavity shows a blueshift of all photonic resonances with increasing angle because of their photonic dispersions. Note that the Bragg modes (BM*n*) show a less pronounced blueshift, which is due to their different photonic confinement.

In order to extract the excitonic and photonic fractions of exciton-polaritons, we determined the Hopfield coefficients *n* by using the following equation

$$\begin{pmatrix} E_{X2} & 0 & V_2 & V_2 & \cdots & V_2 \\ 0 & E_{X1} & V_1 & V_1 & \cdots & V_1 \\ V_2 & V_1 & E_{Ph,1} & 0 & \cdots & 0 \\ V_2 & V_1 & 0 & E_{Ph,2} & \cdots & 0 \\ \vdots & \vdots & \vdots & \vdots & \ddots & \vdots \\ V_2 & V_1 & 0 & 0 & 0 & E_{Ph,n} \end{pmatrix} \begin{pmatrix} \alpha \\ \beta \\ \gamma_1 \\ \gamma_2 \\ \vdots \\ \gamma_n \end{pmatrix} = E_{LPn} \begin{pmatrix} \alpha \\ \beta \\ \gamma_1 \\ \gamma_2 \\ \vdots \\ \gamma_n \end{pmatrix} \quad (5)$$

with E_{X1}, E_{X2} being the excitonic energies, V_1, V_2 the coupling constants, $E_{ph,n}$ the uncoupled photon energies and E_{LPn} the eigenvalues of the matrix. The squares of the entries of the eigenvectors (α, β, γ_1, …, γ_n) are the exciton/photon content for each polariton eigenstate. For our system, we limited the number of cavity modes to $n = 10$.

Figure S3 shows the calculated excitonic fractions α^2 and β^2 as a function of cavity thickness (left) and emission angle (right). With increasing cavity thickness, the cavity modes shift to smaller energies, thus affecting the coupling mechanism. As a consequence, the polariton states also shift to smaller energies. By changing the cavity thickness, the respective polariton eigenvalues in fig. S3 were tuned across the entire spectral range of interest (from 550 nm to 450 nm). As an example, one of the photonic fractions (γ_7^2) is also shown in fig. S3 (black line). The excitonic fractions α^2 and β^2 exhibit two maxima that are related to the excitonic transitions X1 (at 3750 nm) and X2 (at 3600 nm). When tuning the cavity to the optimal thickness for condensation (around 3500 nm in this case), the excitonic fractions are smaller indicating that the polariton condensate has a substantial photonic character. The photon fraction of the condensed polaritons is ~86% at zero emission angle. Increasing the emission angle (fig. S3, right) increases the excitonic fraction of the polariton mode, which is due to the polariton dispersion-induced blueshift of the lower polariton branch and the subsequent crossing of the excitonic transitions X1 and X2.

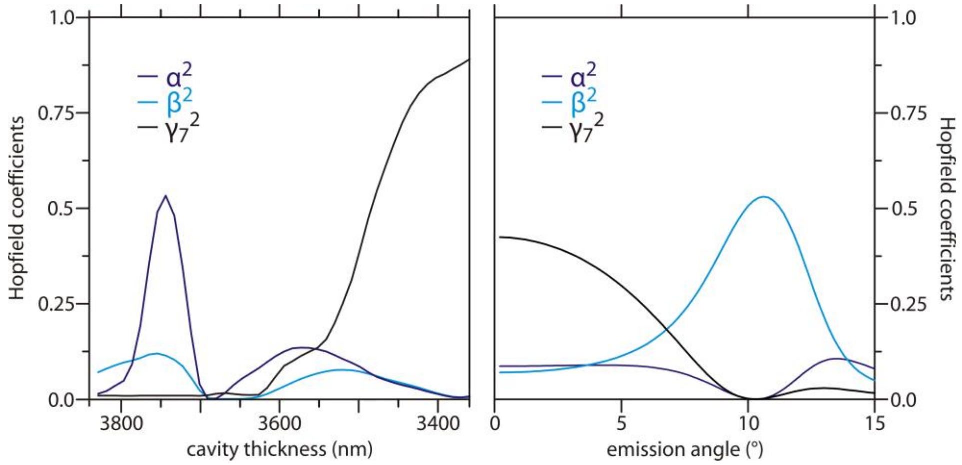

fig. S3. Hopfield coefficients. *Thickness- (left) and angular-dependent (right) calculated Hopfield coefficients* α^2*,* β^2 *and* γ_7^2 *of the microcavity described above. The Hopfield coefficients were calculated by using Eq. 5 with coupling coefficients* V_1 *= 97 meV and* V_2 *= 46 meV. The angular-dependent Hopfield coefficients represent excitonic and photonic fractions of the polariton state LP2.*

sectionS3. Excitation-dependent zero-momentum emission of the eGFP microcavities

Figure S4 shows the non-normalized spectra of the microcavity emission at zero emission angle for different excitation powers. Like the angular-resolved luminescence maps shown in Fig. 3 of the main manuscript, the different spectra show the condensation of the polariton mode LP2 (16 nJ, blue line), the interaction-induced blueshift of the condensate (25 nJ, grey line), the disappearance of the condensate at excitation powers >50 nJ and the subsequent onset of the weak coupling regime (110 nJ, red line) as well as photon lasing (198 nJ, light grey line).

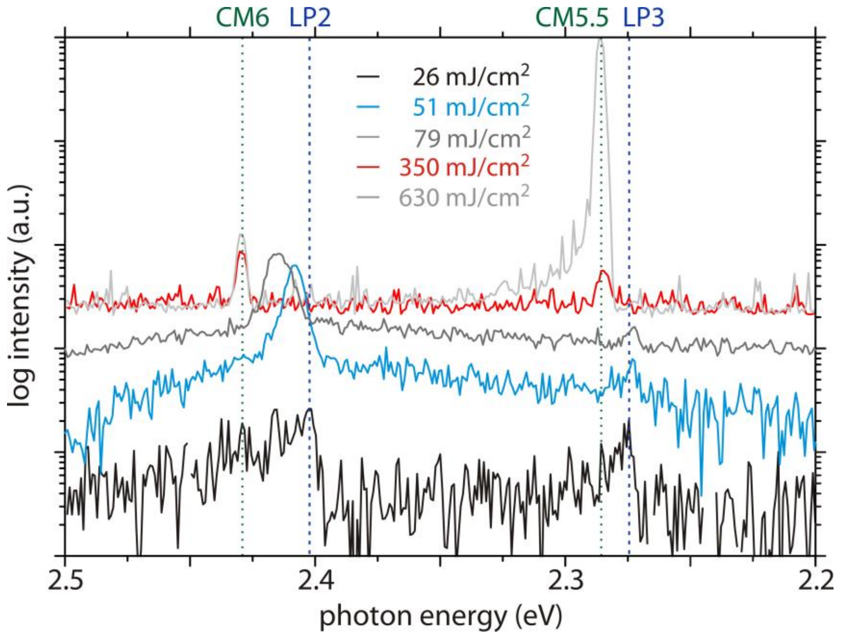

fig. S4. Zero-momentum emission of eGFP-filled microcavity under nanosecond optical excitation. *Excitation powers are given in the upper left corner. The spectral positions of polaritonic (LPn) and photonic (CMn) transitions are indicated by vertical dashed and dotted lines, respectively.*

Note that the derived pump pulse energy threshold value for the onset of polariton lasing (P_1 = 12 nJ) relates to a peak excitation power density of 3.4 MW/cm^2 and an excitation flux of 24 mJ/cm^2 (taking into account an excitation spot of 8 μm and pulse lengths of 7 ns).

section S4. Thermalization of the polariton emission

Figure S5 shows the distribution of the polariton occupancy along the dispersion LP2 for different pump fluences (c.f. Fig. 3 in main text for definition of LP2). The occupancy is corrected for the energy-dependence of the density of states at different momentum values (also accounting for the change of polariton character along the dispersion) and is normalized. The data was then fitted using a Boltzmann distribution

$$I \propto \exp\left(-\frac{E_{LP2} - E_{LP2}(k = 0)}{k_b T}\right)$$

This yielded effective polariton temperatures of 757 K, 373 K and 315 K for excitation densities 0.1 P_1, 0.9 P_1 and 1.0 P_1, respectively. For excitation near the condensation threshold, the determined polariton temperature is close to the room temperature value, meaning that polaritons are fully thermalized. However, we emphasize that even close to condensation threshold the polariton occupancy rather follows a Maxwell-Boltzmann-like behavior than a Bose-Einstein distribution (indicated by the absence of Bose narrowing close to the dispersion minimum). From this, we conclude that our system is in a non-equilibrium state (*31*, *32*). Thermal equilibrium for a polariton condensate was experimentally observed in long-lived microcavities (*33*).

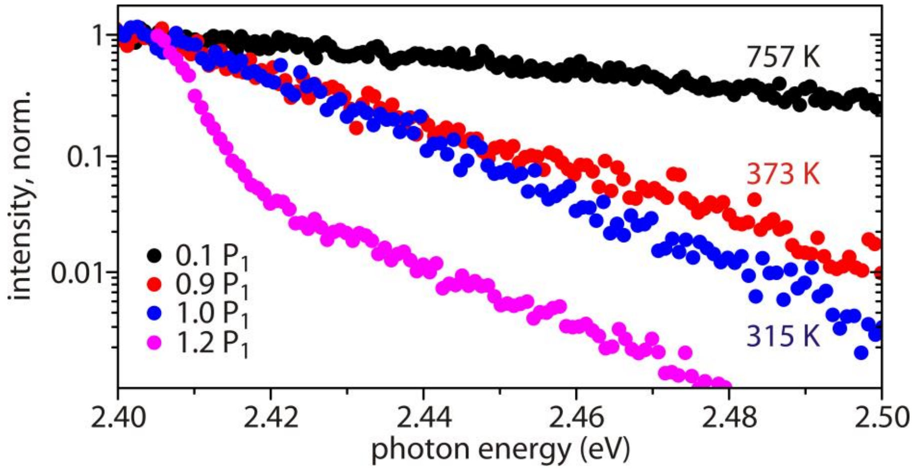

fig. S5. Energy dependence of occupation of the LP2 polariton branch for different excitation densities. *Boltzmann-fits to the data reveal temperatures of 757 K, 373 K and 315 K for excitation densities of 0.1 P_1, 0.9 P_1 and 1.0 P_1, respectively. Above threshold (1.2 P_1), the intensity is drastically enhanced towards to the ground state (at 2.4 eV) while maintaining the same high-energy tail as for P = 1.0 P_1.*

section S5. Polarization of the condensate

Bose-Einstein condensation is accompanied by symmetry breaking which manifests itself in a condensate polarization that is pinned to the polarization of the excitation laser. In order to verify the process of polarization pinning, we examined the polarization-dependent response of the microcavity filled with eGFP under fixed polarization of the excitation beam (parallel to the entrance slit of the spectrometer, 0°→180°). While the microcavity emission shows no polarization-dependence at all in the linear regime ($P < P_1$; fig. S6, left), a clear pinning is observed for the polariton condensate ($P_1 < P < P_2$; fig. S6, right). Since the dipoles of the eGFP fluorophores have a random orientation, only fluorophores with a significant dipole moment parallel to the linearly polarized excitation laser are excited during pumping. As pointed out in Ref. (*10*), depolarization due to Förster energy transfer is much slower than the onset of condensation leading to the preservation of the excitation polarization in the condensate. Note that the polarization dependence in fig. S6 is different from Fig. 3 in the main manuscript where we excited the microcavity with unpolarized light and detected the emission only for TE-polarization.

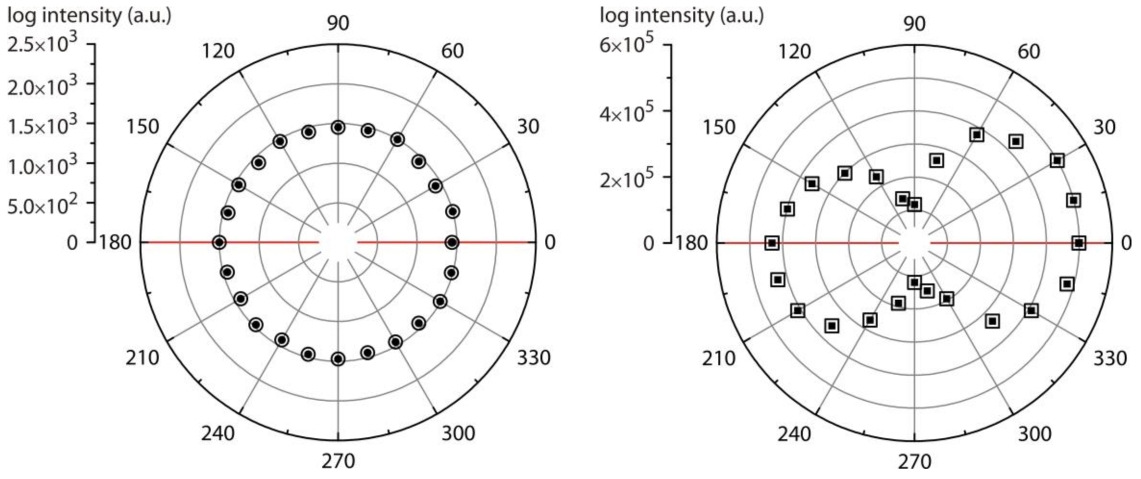

fig. S6. Polarization pinning of the condensate. *Polarization-dependent integrated intensity of the microcavity emission below condensation threshold (left) and above (right). The red line indicates the linear polarization plane of the excitation laser.*

section S6. Spatial coherence of the polariton condensate

We measured the spatial coherence of the polariton condensate by using a Michelson interferometer in mirror-retro-reflector configuration: the real-space luminescence image from the microcavity is split by a 50:50 beam splitter into two arms. One arm is directed towards a 45° retro-reflector flipping the image and reflecting it back to the beam splitter. The other arm is directed towards a plane mirror that is mounted on a piezo-controlled linear translation stage. In this way, the image of the sample and its reversed image interfere with each other at the output of the beam splitter where a high-resolution imaging CCD camera records the incident interference patterns. The position of the plane mirror is tuned to maximize fringe contrast. Examples of the resulting interference patterns are shown in fig. S7, for excitation powers below (A) and well above condensation threshold P_1 but still below photon lasing threshold (B). The images are single-shot measurements (excited by a single 7 ns pulse at 460 nm) and the emission is passed through edge pass filters to only show the 500-600 nm range. The laser spot is indicated by white dashed ellipses in fig. S7 (half the peak power of the Gaussian excitation beam). Whereas the pattern does not show any significant structure below threshold, clear fringes are evident above threshold providing evidence for the presence of coherence across the spatial extend of the condensate. Note that in fig. S7B, the lateral extension of the interference fringes is wider than the excitation spot. Since the system is pumped well above threshold and the dashed line indicates half the peak power of the excitation beam, we assign this to coherence contributions from sample areas that are still pumped above threshold but are outside the marked area. [Given the 0.55 numerical aperture of the used imaging system, we believe that the increase in size is unlikely to reflect a limit of the point-spread function of the imaging system (*34*).] Furthermore, the interference fringes occasionally show fork-like dislocations (see white circle in fig. S7).

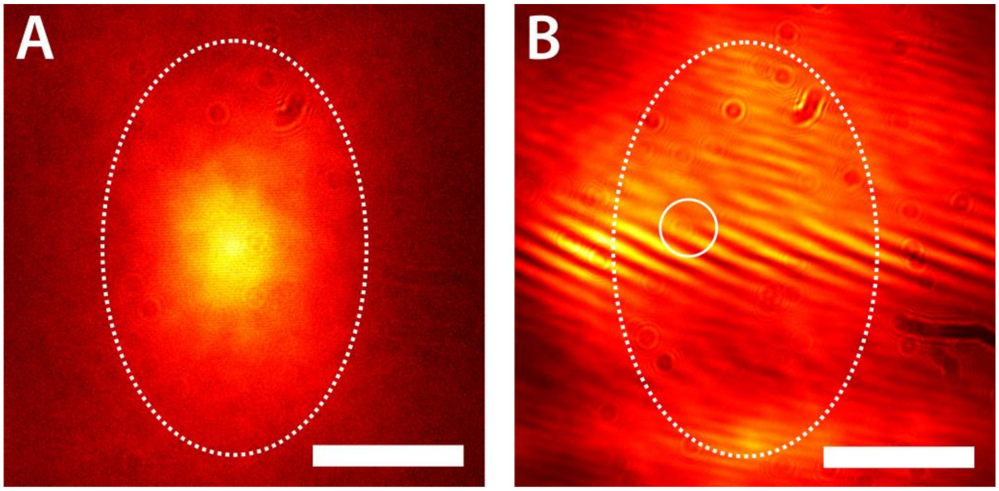

fig. S7. Spatial coherence of eGFP polariton condensates. *Single-shot, real-space Michelson interferograms of an eGFP microcavity excited below (**A**) and above (**B**) condensation threshold P_1. The image is spectrally filtered by using 500 nm long-pass and 600 nm short-pass filters. The white circle in the right image indicates a fork-like dislocation of the interference fringes. Scale bars, 5 μm.*
